# Artificial Intelligence in the Diagnosis and Treatment of Brain Gliomas

**DOI:** 10.3390/biomedicines13092285

**Published:** 2025-09-17

**Authors:** Kyriacos Evangelou, Ioannis Kotsantis, Aristotelis Kalyvas, Anastasios Kyriazoglou, Panagiota Economopoulou, Georgios Velonakis, Maria Gavra, Amanda Psyrri, Efstathios J. Boviatsis, Lampis C. Stavrinou

**Affiliations:** 1 Second Department of Neurosurgery, Faculty of Medicine, National and Kapodistrian University of Athens, Attikon University Hospital, 12462 Athens, Greece; aristoteliskalyvas@gmail.com (A.K.); eboviatsis@gmail.com (E.J.B.); lampis.stavrinou@gmail.com (L.C.S.); 2 Section of Medical Oncology, Second Department of Internal Medicine, Faculty of Medicine, National and Kapodistrian University of Athens, Attikon University Hospital, 12462 Athens, Greece; ikotsantis@gmail.com (I.K.); tassoskyr@gmail.com (A.K.); panagiota_oiko@hotmail.com (P.E.); psyrrioncology@gmail.com (A.P.); 3 Division of Neurosurgery, Department of Surgery, Temetry Faculty of Medicine, University of Toronto, Toronto, ON M5T 2S8, Canada; 4 Second Department of Radiology, Faculty of Medicine, National and Kapodistrian University of Athens, Attikon University Hospital, 12462 Athens, Greece; giorvelonakis@gmail.com; 5 Department of CT and MRI, ‘Agia Sofia’ Children’s Hospital, 11527 Athens, Greece; mmgavra@yahoo.com

**Keywords:** artificial intelligence, brain tumor, glioma, glioblastoma, neurosurgery, oncology

## Abstract

Brain gliomas are highly infiltrative and heterogenous tumors, whose early and accurate detection as well as therapeutic management are challenging. Artificial intelligence (AI) has the potential to redefine the landscape in neuro-oncology and can enhance glioma detection, imaging segmentation, and non-invasive molecular characterization better than conventional diagnostic modalities through deep learning-driven radiomics and radiogenomics. AI algorithms have been shown to predict genotypic and phenotypic glioma traits with remarkable accuracy and facilitate patient-tailored therapeutic decision-making. Such algorithms can be incorporated into surgical planning to optimize resection extent while preserving eloquent cortical structures through preoperative imaging fusion and intraoperative augmented reality-assisted navigation. Beyond resection, AI may assist in radiotherapy dose distribution optimization, thus ensuring maximal tumor control while minimizing surrounding tissue collateral damage. AI-guided molecular profiling and treatment response prediction models can facilitate individualized chemotherapy regimen tailoring, especially for glioblastomas with MGMT promoter methylation. Applications in immunotherapy are emerging, and research is focusing on AI to identify tumor microenvironment signatures predictive of immune checkpoint inhibition responsiveness. AI-integrated prognostic models incorporating radiomic, histopathologic, and clinical variables can additionally improve survival stratification and recurrence risk prediction remarkably, to refine follow-up strategies in high-risk patients. However, data heterogeneity, algorithmic transparency concerns, and regulatory challenges hamstring AI implementation in neuro-oncology despite its transformative potential. It is therefore imperative for clinical translation to develop interpretable AI frameworks, integrate multimodal datasets, and robustly validate externally. Future research should prioritize the creation of generalizable AI models, combine larger and more diverse datasets, and integrate multimodal imaging and molecular data to overcome these obstacles and revolutionize AI-assisted patient-specific glioma management.

## 1. Introduction

Gliomas are the most common primary brain tumors (30–40% of all intracranial tumors) and originate from glial cells [1]. They include a spectrum of subtypes with distinct histopathological and molecular characteristics, with grade IV gliomas and glioblastomas (GBM) accounting for approximately 50% of cases [2]. The World Health Organization (WHO) classifies gliomas into four grades (I–IV) based on a combination of histological features (e.g., cellular atypia, mitotic activity, necrosis) and molecular genetic markers. Key genetic alterations used for classification include isocitrate dehydrogenase 1 (IDH1) mutations, 1p/19q codeletion, telomerase reverse transcriptase (TERT) promoter mutations, epidermal growth factor receptor (EGFR) amplification, and the concurrent gain of chromosome 7 with loss of chromosome 10 (+7/−10) [3]. Grade I tumors (e.g., pilocytic astrocytomas) are typically benign and more frequently encountered in the pediatric population, while grade IV tumors are highly aggressive and predominantly affect adults [4].

The incidence rate of gliomas reaches around six per 100,000 individuals globally and is higher in Europe and North America [5]. The etiology of glioma development is multifactorial and definite causative factors have yet to be fully elucidated, although genetic predispositions and environmental influences are speculated to participate in tumorigenesis [6]. Molecular genetic advancements have remarkably improved glioma pathogenesis understanding and have laid the groundwork for targeted therapeutic strategies and personalized treatment plans [7].

The management of brain gliomas faces significant challenges; traditional diagnostic modalities such as computed tomography (CT) and magnetic resonance imaging (MRI) often lack the necessary sensitivity to detect subtle tumor characteristics that would expedite accurate diagnosis, a challenge exacerbated by the limited accessibility to advanced diagnostic tools in certain regions [8]. Treatment is further complicated by the inherent molecular heterogeneity of gliomas, with genetic and epigenetic profile variations precipitating volatile therapeutic responses. This exact variability contributes to inconsistent treatment outcomes across different cases [9].

Monitoring glioma progression remains challenging due to the diffuse infiltration of the surrounding healthy brain tissue that hinders the accurate differentiation between tumor recurrence and treatment-associated changes [10]. The blood–brain barrier (BBB) itself represents an additional treatment obstacle by restricting central nervous system (CNS) chemotherapeutic drug delivery [11]. Efflux transporters such as P-glycoprotein (P-gp) and breast cancer resistance protein (BCRP) can impede the penetration of standard chemotherapeutic agents such as temozolomide (TMZ) and reduce their concentration at the tumor site, limiting their efficacy [12]. Furthermore, intrinsic tumor resistance mechanisms such as DNA repair enzyme expression (e.g., O [6]-methylguanine-DNA methyltransferase; MGMT) further diminish TMZ effectiveness and underscore the need for alternative therapeutic approaches in an effort to improve glioma prognosis [13].

The implementation of Artificial Intelligence (AI) has the potential to advance radiological imaging and personalized treatment in oncology [14]. Automated image analysis using AI-driven algorithms has been shown to improve diagnostic accuracy by identifying MRI or CT patterns that might often elude human observation, facilitating both early disease detection and precise tumor characterization [15]. AI-mediated imaging segmentation using semi- or fully automated methods can not only improve the high-precision delineation of tumor boundaries but also encourage the development of personalized treatment plans tailored to the unique radiological tumor morphology of each patient [16]. AI-powered predictive analytics enable informed decisions by assessing potential treatment outcomes, while real-time monitoring of treatment responses allows for timely adjustments to such interventions [17]. The integration of multi-omics data (encompassing genomic, proteomic, and metabolomic information) into AI platforms facilitates treatment customization even further [18]. Finally, AI can optimize dose distribution in radiation therapy planning by accurately targeting diseased regions while sparing healthy tissue [19].

**Figure 1:**
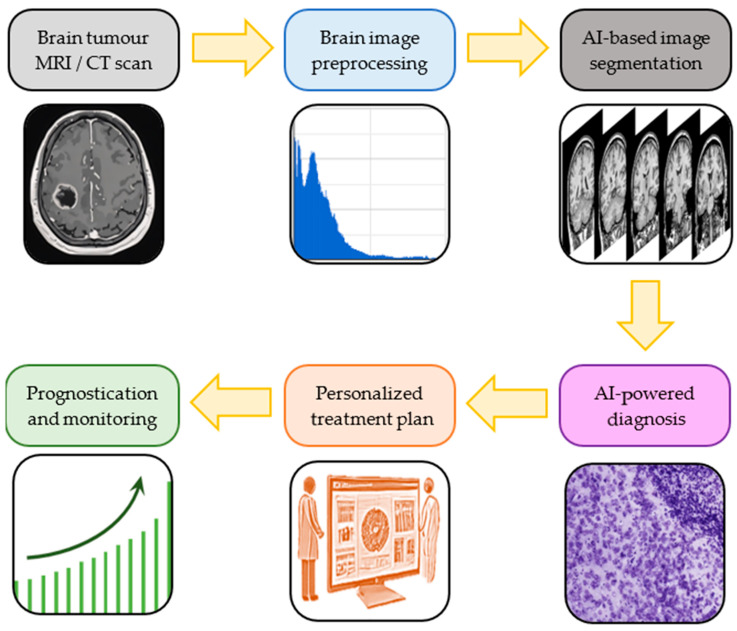
Artificial intelligence (AI) workflow sequential steps in brain glioma diagnosis and treatment. Multimodal imaging-derived brain tumour images are preprocessed and segmented with the help of AI; this facilitates accurate diagnosis and the development of personalized treatment plans, which improve patient prognostication and monitoring.

The purpose of this review is to serve as a comprehensive analysis of the current and emerging applications of AI in glioma diagnosis and treatment, highlighting how AI-powered technologies can enhance diagnostic precision, facilitate personalized therapeutic strategy development, and improve prognostic assessments in disease management (Figure 1).

This perspective on the current utility and potential of AI in neuro-oncology ultimately aims to inform clinicians and researchers about the groundbreaking footprint of AI on glioma management and identify avenues for future research and further integration of advanced technologies into clinical practice.

## 2. Fundamentals of AI in Medical and Neuro-Oncology

### 2.1. Overview and History of AI Technologies

**Figure 2:**
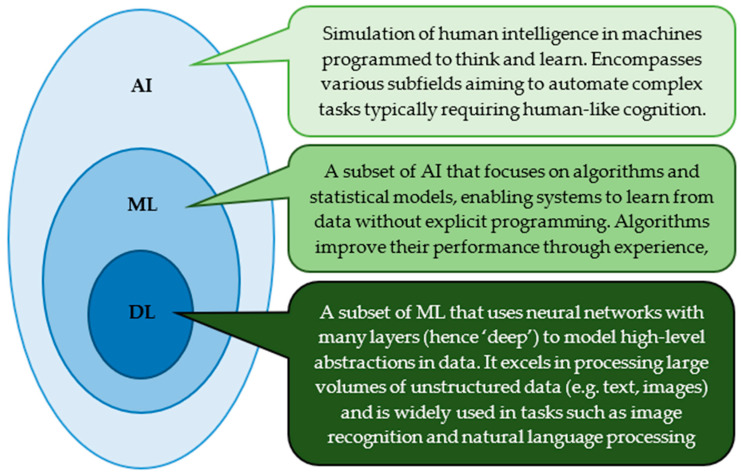
Hierarchical relationship between Artificial Intelligence (AI), Machine Learning (ML), and Deep Learning (DL). AI is the broadest concept, encompassing ML, which in turn contains DL as a more specialized subset focused on complex data processing through neural networks.

Artificial intelligence encompasses multiple computational methodologies (e.g., pattern recognition, decision-making, problem solving) that allow the implementation of tasks that conventionally prerequisite human intelligence [20]. One of the most important subfields of AI is machine learning (ML), which involves algorithms that learn from data and are able to make predictions or decisions sans explicit programming [21]. Complex data patterns can be modelled using artificial neural networks with multiple layers by a prominent subset of ML known as deep learning (DL) (Figure 2) [22].

DL has advanced image analysis in neuro-oncology and enhanced both diagnostic accuracy and prognostic assessments [23]. One example is the application of DL in the automation of both bidimensional and volumetric tumor burden measurement from MRI in GBM patients with comparable accuracy to board-certified neuroradiologists [24]. DL techniques have also been implemented for the direct prediction of molecular alterations from histopathological slides in an effort to characterize brain tumors non-invasively [25].

**Figure 3:**
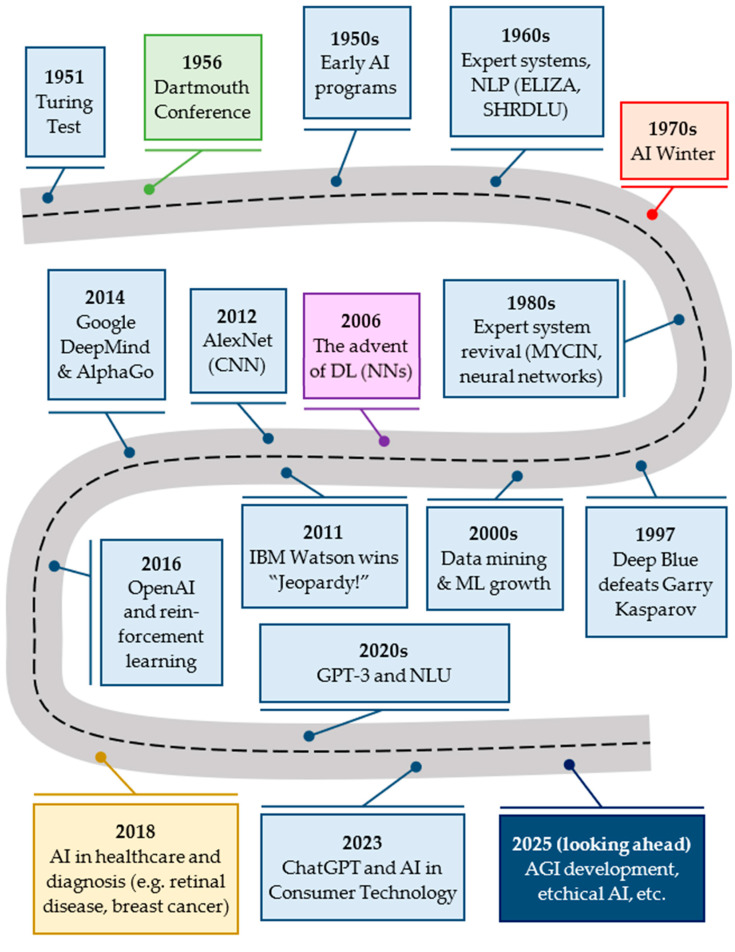
Timeline of key milestones in AI development from its inception in the 1950s to the groundbreaking advancements of the 2020s [33]. The Turing Test was proposed by Alan Turing to determine if a machine could exhibit human-like intelligence, something which later became a foundational concept in AI. Deep Blue of the International Business Machines Corporation (IBM) was a chess-playing computer that defeated world champion Garry Kasparov, marking a significant achievement for AI in strategy and game-playing. Watson is a computer system that defeated human champions in the quiz show “Jeopardy!”, showcasing advancements in natural language processing (NLP) and AI’s ability to understand and respond to complex questions. AlexNet was a convolutional neural network (CNN) that won the ImageNet competition by a large margin, revolutionizing the field of computer vision. In the present environment, efforts to develop Artificial General Intelligence (AGI) continue to progress, with a focus on creating AI that can understand, learn, and apply knowledge in a human-like manner across diverse tasks. Ethical considerations, including AI governance and fairness, will play an increasingly significant role as AI becomes more integrated into daily life. AI = artificial intelligence; DL = deep learning; GPT-3 = generative pre-trained transformer 3; ML = machine learning; NLU = natural language understanding.

Significant milestones have marked the evolution of AI in medicine since its inception; the foundation for AI research had been laid during the Dartmouth Conference in 1956 with the coining of the term “artificial intelligence” [26]. AI gradually began to demonstrate its potential in the medical field in the early 1970s; systems like INTERNIST-1 were developed in 1971 as the world’s first artificial medical consultant that could diagnose patients based on their symptoms through the utilization of algorithms [27]. The MYCIN system was similarly initiated at Stanford in 1972 and could identify bacterial infections and recommend appropriate treatments via AI-powered methods [28]. Despite their advancements, early AI models faced multiple limitations that hindered their widespread adoption across the medical community of the era, the so-called first (1974–1980) and second (1987–1994) AI winters [29]. The dawn of the second millennium saw a resurgence in the medical applications of AI that was primarily driven by the advent of DL techniques capable of complex algorithmic analysis and self-learning and culminated in the streamlining of its diagnostic accuracy and workflow efficiency [30]. The transitional period of the last two-and-a-half decades constituted an upsurge in the capabilities and development of AI with an abundance of significant historical landmarks; by 2025, the integration of AI into healthcare has reached unprecedented levels, including the detection of psychiatric disorders (e.g., schizophrenia) [31] and the execution of autonomous laparoscopic surgeries without human intervention [32]. Figure 3 highlights pivotal moments in AI history, showcasing significant achievements, periods of decline, and the emergence of transformative technologies in ML and DL.

### 2.2. Radiomics and Radiogenomics

Radiomics is a rapidly evolving method of voluminous data extraction from radiological images using data characterization algorithms that can transform complex qualitative data into quantifiable, reproducible, and analyzable features [34]. These quantitative metrics, obtained through advanced computational algorithm application to MRI or CT scans, can be used to extract clinically relevant information for glioma diagnosis and treatment and characterize tumor biological behavior, morphology, and microenvironment with capabilities far superior to what the human eye can achieve [35]. Key examples include the quantification of texture, shape, and signal intensity features to reveal subtle variations in tissue properties within the tumor microenvironment that reflect cellularity, necrosis extent, and glioma vasculature [36,37]. Heterogeneity features such as co-occurrence matrices, entropy and contrast are known to correlate with histopathological features (e.g., grade, subtype) in gliomas and can be utilized to facilitate prompt diagnosis by detecting early tumor changes or visible radiological alterations that may precede clinical symptom manifestation [38,39].

Non-invasive lesion characterization is another notable application of radiomics in glioma diagnosis; techniques such as diffusion-weighted imaging (DWI) or perfusion MRI can be employed to extract features indicative of tissue architectural characteristics (e.g., fractional anisotropy and apparent diffusion coefficient) that differentiate low- from high-grade lesions [40,41]. The radiomics-mediated identification of tumor-specific abnormal vascularization patterns or perilesional oedema visualized through dynamic contrast-enhanced MRI can further assist disease progression assessment and post-therapeutic recurrence prediction [42], potentially guiding surgical planning, monitoring therapeutic responses real-time, and enabling timely intervention by tracking changes that may precede clinical relapse.

Radiogenomics represents the integration of the aforementioned radiomics with genomic and molecular data, linking imaging phenotypes with genetic and molecular tumor characteristics such as biomarkers traditionally determined through invasive tissue sampling [43]. Specific imaging phenotypes including tumor texture patterns, apparent diffusion coefficient (ADC) values and the degree of contrast enhancement have been found to correlate with molecular subtypes; features indicative of a more aggressive tumor phenotype (e.g., increased necrosis, irregular vascular patterns) often correspond to gliomas with genetic alterations in key oncogenes and tumor-suppressor genes, such as the isocitrate dehydrogenase (IDH)-status, tumor protein 53 (TP53) mutations, and EGFR amplification [44]. Especially regarding IDH-wildtype and IDH-mutant gliomas, DL algorithms are becoming increasingly capable of identifying their status, as the former tend to demonstrate more aggressive features such as solid enhancing regions and necrotic core formation, while the latter often present with less prominent contrast enhancement and diffuse oedema on MRI, sometimes mimicking low-grade gliomas [45,46].

There are also indications that the presence of specific mutations like the TERT promoter mutations may influence imaging characteristics such as tumor location, cortical extension, and peritumoral tissue response [47]. These mutations increase EGFR amplification and interleukin 6 (IL-6) levels, inducing angiogenesis and tumor necrosis [48], and decrease glycogen accumulation, limiting glucose oxidation, nucleic acid and de novo fatty acid synthesis, and promoting tumor cell apoptosis [49]. TERT promoter mutations have been associated with increased cortical extension and necrosis, as well as specific tumor locations (more common than their wild-type counterparts in the insula and hemispheric lobes but rarer in the basal nuclei, thalamus, brainstem, and cerebellum) [47]. Radiogenomics can thereby bridge the gap between imaging-based diagnosis and genetic-based treatment approaches to aid pre-biopsy decision-making, especially when it comes to surgical planning, and particularly in regard to the extent and aggressiveness of resection.

### 2.3. AI Algorithms Commonly Used in Neuro-Oncology

**Figure 4:**
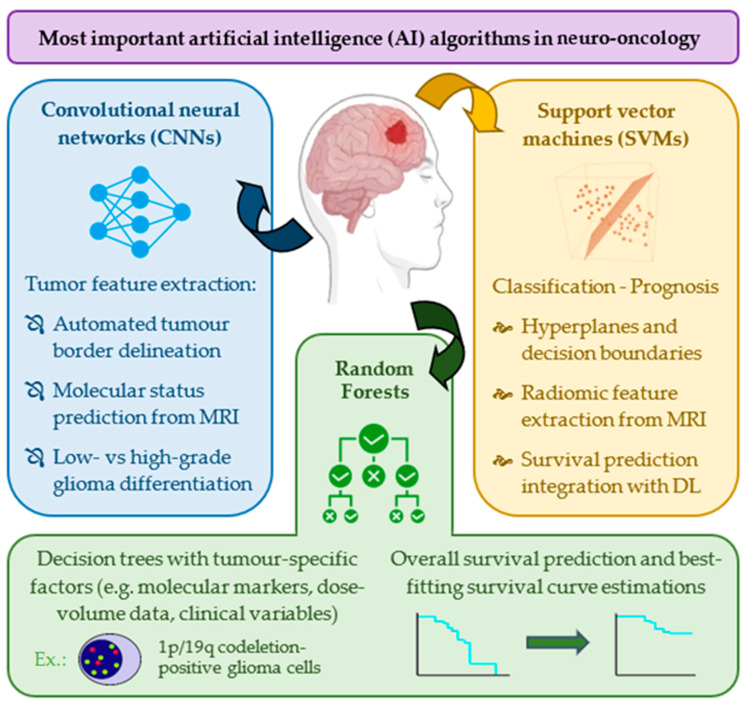
Artificial intelligence (AI) algorithms in neuro-oncology for glioma diagnosis, prognosis assessment, and treatment facilitation. 1p = short arm of chromosome 1; 19q = long arm of chromosome 19; DL = deep learning; MRI = magnetic resonance imaging.

Several AI algorithms have been employed in neuro-oncology to enhance diagnostic accuracy, prognostic assessments and treatment planning of gliomas, with Convolutional Neural Networks (CNNs), Support Vector Machines (SVMs), and Random Forests demonstrating documented utility (Figure 4).

CNNs are a class of DL models adept at processing imaging and other structured grid data to automatically extract features [50]; they have been used to differentiate between low- and high-grade gliomas [51], accurately delineate tumor boundaries through MRI segmentation [52], or even predict molecular markers such as MGMT promoter methylation status from MRI scans [53]. SVMs are supervised learning models that classify data through the identification of the optimal hyperplane (i.e., decision boundary) separating different classes [54]; these have been used to both differentiate between glioma grades based on MRI-extracted radiomic features [55] and predict overall survival through integration with DL architectures [56]. Finally, Random Forests construct multiple decision trees during training and output the class mode or mean prediction for classification and regression tasks, respectively [57]; radiomics signatures developed using Random Forest classifiers can predict molecular markers such as the 1p/19q codeletion status [58], while ML Random Forest models incorporating clinical variables and dose-volume histogram parameters have managed to outperform other models (Cox proportional hazards and even SVMs) in both training and testing sets of high-grade glioma overall survival prediction studies [59].

## 3. Applications of AI in Glioma Diagnosis

### 3.1. Lesion Detection and Imaging Segmentation

Although hybrid models combining CNNs and Vision Transformers (ViT; DL architectures that process images via self-attention mechanisms) can effectively capture both global and local features from MRI images to improve glioma classification accuracy [60], AI can analyze scans from imaging modalities beyond conventional MRI and CT, such as Positron Emission Tomography (PET) and Magnetic Resonance Spectroscopy (MRS). The last quinquennium has provided insights that AI models can ground on the former to differentiate between pseudoprogression and true progression [61,62], radionecrosis and tumor recurrence [63,64] and low- and high-grade lesions [65]. There is growing evidence that AI can interpret MR spectra and identify key metabolites that are critical in glioma detection, such as N-acetylaspartate (NAA), choline (Cho), creatine (Cr), and lactate (Lac) [66]. ML models successfully differentiated normal-appearing from cancerous white matter and future disease progression in a 2024 study with 16 high-grade glioma patients and recognized the Cho/Cr ratio as the most important feature; the authors remark that these advanced technological models could demystify complex metabolite relationships and unveil radiologically occult glioma progression in post-treatment tumors up to eight months in advance in their first-of-its-kind study [67]. Previous reports documented SVM classifiers capable of predicting diffuse glioma grade in both training (area under curve (AUC) 0.825) and validation sets (AUC 0.820) in humans [68] and differentiating between TMZ-treated and -untreated mice with GBM with an accuracy of over 80% [69] based on pre- and/or postoperative MRS images.

The previously introduced tumor border delineation has probably been one of the most anticipated potentials of novel AI-powered technologies; a minimally invasive, non-ionizing, contrast-free process called hyperspectral imaging (HSI) has emerged as an intraoperative guidance technique for chemocomposition-based tissue identification stemming from a continuous pixel-specific spectrum [70]. A trinational collaboration (Spain; United Kingdom; France) has developed, extended, and improved several novel algorithms for brain tumor classification grounded in their spatiospectral characteristics. The initial algorithm underwent training on a labelled dataset of over 300,000 spectral signatures of the visible and near-infrared (VNIR) range (400–1000 nm) and could generate thermatic maps which could easily differentiate between normal tissue, tumor tissue, blood vessels/hypervascularized tissue, and their background with an accuracy of over 99% [71]. Fusing multiple maps obtained from five different grade IV (GBM) patients generated a final classification map that could subsequently delineate the boundaries of the tumors [72]. Although further application determined a specificity of 100% with nihil false positives, the authors recognize that postresectional effects such as blood extravasation, normal peritumoral tissue vascularization augmentation, and electrocautery marks predispose to misclassification errors [73]. The team extended their dataset to generate and validate their classification results more robustly through a more exhaustive spectral characteristic identification across tissue types [74].

To achieve tumor border delineation and similar processes associated with accurate preoperative tumor characterization and localization, it is often necessary to properly segment MRI images. Although manual techniques used to be the gold standard for precise segmentation [75], they remain tremendously time-consuming and introduce a grade of outcome variability among different -even highly experienced- neuroradiologists [76,77]. Multiple software have been developed for automated segmentations with similar -and often superior- results to manual segmentation; an example is the Brain Tumor Image Analysis (BraTumIA) software, which utilizes automated volume measurements comparable to manual delineation for contrast-enhanced tumor volumes and superior interrater variability regarding overlap and sensitivity [78].

Promising yet suboptimal results with interobserver variability among experts were demonstrated a decade ago regarding semi-automated high-grade glioma segmentation [79], however significant leaps have been attained by virtue of technological advancements; In 2024, fine-tuned DL models can automatedly segment diffuse-type gliomas with sufficient Dice Similarity Coefficients (DSC; a spatial overlap index and a reproducibility validation metric) to radiologists, especially for newly diagnosed (*p* < 0.001) and unifocal (*p* = 0.001) tumors [80]. State-of-the-art CNN architectures for GMB segmentation trained and validated on cohorts recruiting almost 1,000 patients have performed comparably well to clinicians in gross total resection versus residual tumor patient distinction [81]. Although non-manual segmentation methods can improve efficiency and increase the reproducibility of results, there is still a need for further development and amelioration of the current algorithms to refine the existing models through manual segmentation-based training [82].

### 3.2. Differential Diagnosis of Gliomas

Building upon its role in glioma segmentation, the capabilities of AI extend to differentiating gliomas from other common intracranial tumors. A 2022 systematic review and meta-analysis (SRMA) revolving around ML methods to differentiate between gliomas and primary central nervous system lymphomas (PCNSLs) included 23 articles and more than 2,000 patients; Logistic regression and SVM models using conventional radiomic features accomplished a topmost AUC of 96.1% with an externally validated accuracy of 91.2% [83]. The between-study heterogeneity was nevertheless high in the meta-analysis as the studies varied in terms of ML model pipelines. Another 2022 SMRA focusing specifically on GMB differentiation from PCNSL on MRI only included ten studies (sample size: 1,311) but engaged both ML (60.0%) and DL (40.0%) approaches; when directly compared with the gold standard of differential diagnosis (histopathology), the model sensitivity, specificity, and balanced accuracy spanned 80–99%, 87–100%, and 84–100%, respectively [84]. However, reliability and generalizability of the conclusions were complicated by inherent variations in imaging protocols, MRI machines and sequences, segmentation techniques, and training classifiers across studies.

A third SRMS on glioma differential diagnosis from other intracranial pathologies was published in 2022 focusing on the glioma vs. metastasis conundrum; The authors included 29 studies and investigated model design, development and best classifiers [85]. Pooled accuracy, AUC, sensitivity, and specificity accuracies reached 88.1%, 91.6%, 86.8%, and 84.3%, respectively, while no differences between conventional and advanced MRI were noted in a subgroup AUC analysis. SVM performed the best among the top classifiers (AUC = 93.6%), however more than 85% of all information was derived from single-center institutions and external validation was performed in less than 7% of studies. Furthermore, the overall quality of reporting was deemed insufficient according to the Transparent Reporting of a multivariable prediction model for Individual Prognosis Or Diagnosis (TRIPOD) statement (median adherence = 48%) despite the promising classification results. The authors focus on the limited datasets, poor study design reporting and lack of algorithm validation to highlight the significance of adhering to quality guidelines, validating on external—ideally multi-center—datasets, and sharing algorithms to optimize the clinical application of AI-based models for glioma and metastasis distinction. With time, superior algorithms are being developed that indicate not only that ML and DL can differentiate between the above three intracranial malignancies robustly and comparably well, but also that the peritumoral tissue—aside from the tumor itself—is important for the refinement of such models [86].

A very interesting method of differential diagnosis between GBM and brain metastases using ML and MRI lies in the so-called “oxygen metabolic radiomics”. Combining 1D-CNN with radiomic features from the cerebral metabolic rate of oxygen (CMRO_2_) or tissue oxygen saturation (mitoPO_2_) can surpass radiologists in terms of diagnostic performance [87]. The oxygen extraction fraction (OEF) in contrast-enhancing tumor (CET) areas is lower for GBM, the cerebral blood flow (CBF), OEF and CMRO_2_ are higher in CET compared to non-enhancing T2 hyperintense (NET2) regions for metastases only, while they demonstrate higher CET/NET2 ratios for CBF and CMRO2 than GBM [88]. The rationale behind oxygen metabolism assessment is corroborated by the greater oxygen demand attributable to the higher proliferation rates of GBM that engender dissimilarities in aerobic glycolysis, oxidative phosphorylation, and intratumoral tissue hypoxia compared to cerebral metastases [89].

### 3.3. Non-Invasive Molecular Characterization

Non-invasive approaches for predicting molecular markers such as IDH-mutations and MGMT promoter methylation have been in the forefront of AI-applications. Unlike conventional histopathology reliant on invasive tissue sampling, AI-powered models deploy imaging data to infer molecular alterations [90].

IDH-mutations are present in 70–80% of lower-grade gliomas and signify a more favourable prognosis compared to their wild-type counterparts [91]. Multiple studies have investigated the potential of radiomics in predicting grade II-III glioma IDH-mutation status before and after the recent reclassifications by the WHO in 2016 and 2021 [92,93,94,95,96,97,98]. More recent (2021–2022) same-team studies have tapped into The Cancer Genome Atlas (TCGA) and The Cancer Imaging Archive (TCIA) for the development, external testing, and training of their models, although the datasets were nonetheless small-sized, with a total of 77 (28 wild-type) [99] and 63 (14 wild-type) [100] cases, respectively. Multicentric MRI data from a pool of 142 (46 wild-type) glioma patients were used in 2024 for the development of a radiogenomics model that achieved AUC, sensitivity, and specificity all ≥ 70% in both test cohorts and was additionally deemed unbiased for sex and age following fairness assessment [101]. The synergy of DL with dynamic susceptibility contrast (DSC) MRI was also confirmed in 2024 through an interesting study involving relative cerebral blood volume (rCBV) characteristics; pre-trained and attention gate-enhanced CNNs managed to outperform classical ML models in all outcome components not merely for IDH-mutated, but for TERT gene promoter (TERTp)-mutated gliomas as well [102].

Expectedly, MGMT promoter methylation has also been studied for non-invasive detection, being a key predictor of GBM response to TMZ therapy. Initially, studies have focused on modalities such as ^18^F-fluorodeoxyglucose (FDG) [103] or ^18^F-DOPA (3,4-dihydroxy-6-[^18^F]fluoro-L-phenylalanine) [104] PET and produced significantly accurate results. Even though the same applies to MRI-based predictions using ADC and relative CBF values [105,106], all aforementioned strategies are objectively unfeasible in low-resource institutions with limited access to cutting-edge technologies. Insights from one of the most recent studies utilizing an AI tool for MGMT methylation status prediction based on merely two conventional MRI sequences can therefore be considered at least promising, if not groundbreaking; the authors demonstrated that integrating clinical and radiotherapy derived AI-driven phenotypes based on readily available diagnostic T1-weighted gadolinium-enhanced (T1GD) and T2-FLAIR (FLuid-Attenuated Inversion Recovery) MR images from predefined regions of interest (ROIs) can facilitate noninvasive molecular characterization with high accuracy (82%; AUC = 81%) and precision (75%), even in resource-deficient settings [107]. In evaluating these accuracy percentiles, one should keep in mind that the methods and optimal cutoff definitions for MGMT status determination remain controversial even in the classical setting.

Potent AI-frameworks have been developed not only for preoperative glioma characterization, but also to complement conventional histopathological assessments regarding subtyping, grading, molecular marker prediction and survival prediction [108]. The majority of studies centre around hematoxylin-eosin-stained whole-slide images (WSIs) of diffuse adult-type gliomas, relied on TCGA datasets, and harnessed the potential of CNNs. Certain ML models prerequisite ROI predetermination (often laborious) and WSI-ROI partition into smaller tiles; they are thereupon trained via weakly supervised learning (WSL) and a subtype of it called multiple-instance learning (MIL), which can also function independently from ROIs. Literature reports accuracies ranging from 70% to 100% regarding subtype prediction. Similar results have been observed for grading; however, it is interesting to note that ML models are capable of distinguishing between grade II/III and IV lesions much more accurately than grade II and III ones [109,110,111]. Although most models developed focus on IDH-mutation prediction, studies have also been directing their attention to other markers such as 1p/19q codeletion, homozygous deletion of the cyclin-dependent kinase inhibitor 2A/B (CDKN2A/B), alpha-thalassemia/mental retardation X-linked (ATRX) mutation, EGFR amplification, TP53 mutation, capicua transcriptional repressor (CIC) mutation, etc., since last year with quite promising results [25,112]. Survival prediction is regarded as the most challenging task due to the interplay of multiple factors; past attempts have therefore revolved around graph CNNs, multiple feature detectors (both unimodal approaches), multi modal approaches through the integration of clinical or omics data, and even distinct unimodal feature representation fusions [113,114,115].

## 4. AI-Assisted Therapeutic Planning

### 4.1. Surgical Planning and Intraoperative Assistance

Accurate preoperative planning is critical for ensuring glioma surgery success, given the infiltrating nature of these tumors and their potential proximity to eloquent brain regions [116] such as the primary motor and somatosensory cortex, the visual and auditory cortex, and the cortical and subcortical language networks [117]. Diffusion-tensor imaging (DTI) MR techniques have been utilized for preoperative surgical planning through tractography-based 3D reconstruction that enables the assessment of normal, displaced, or infiltrated white matter tracts, thus increasing both resection extent and function preservation [118]. Regarding supratentorial glioma resection, for example, 3D-reconstruction-based simulatory training using the RadiAnt software (Medixant. RadiAnt DICOM Viewer [Software]. Version 2021.1. 27 June 2021) [119] has already been implemented; such volume rendering-based efforts are economical, user-friendly, easy-to-learn, and can even be used as image-guiding intraoperative techniques [120]. In 2023, a minimal resting-state functional MRI (RS-fMRI)-based 3D-CNN for mapping language and motor data was introduced by a multi-institutional American team and showed an impressive 96% out-of-sample validation accuracy alongside a robust 97.9% mapping similarity with 50 and 200 RS-fMRI time-points, reducing scanning times and improving preoperative planning [121]. Furthermore, it is impressive that ML algorithms have also been developed for the selection of cranial approaches depending on patient-specific anatomical characteristics in neurosurgical oncology [122] to preoperatively aid in intraoperative navigation.

**Figure 5:**
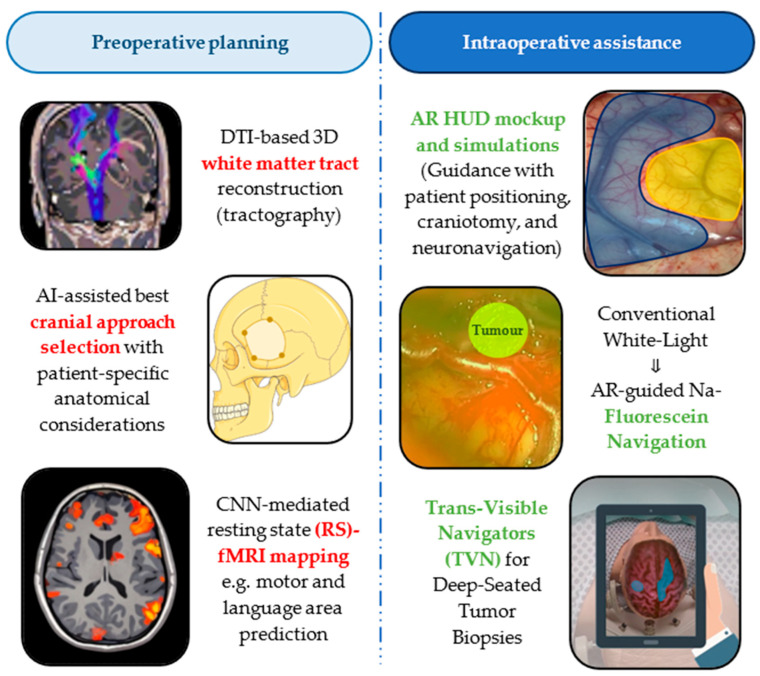
Applications of artificial intelligence (AI) derivatives in postoperative planning and intraoperative assistance for glioma surgery. AR = Augmented reality; CNN = Convolutional neural network; DTI = Diffusion tensor imaging; fMRI = functional magnetic resonance imaging; HUD = heads-up display.

Real-time intraoperative navigation is equally critical and a breakthrough in AR-assisted glioma resection. Head-up displays (HUD) can help initiate surgery, starting from patient positioning, skin incision, craniotomy, and dural opening [123]. There are indications that multimodal augmented reality (AR) high-definition fiber tractography with sodium fluorescein (HDFT-F)-based cytoreductive surgery might be superior to conventional white-light infrared neuronavigation-assisted surgery [124], while the development of novel AR navigation systems is ongoing: a tablet-based system called “trans-visible navigator (TVN)” has been successfully tested for deep-seated tumor stereotactic biopsies, facilitating target point accuracy and trajectory suitability confirmation [125], as well as glioma resection, especially for superficial lesions and those removed via a transcortical or interhemispheric approach [126]. Even mobile AR (mAR) has been proposed as a low-cost alternative; iPhone-compatible mAR was found to be technically feasible with fast (<10 min.) image projection simplification and accuracy comparable to frameless neuronavigation [127], while apps like SINA (Sina Intraoperative Neurosurgical Assist) [128] achieved practicality and reliability over conventional neuronavigation for supratentorial glioma localization [129]. AR can also guide neuroendoscopy to optimise burr hole placement, tumor location estimating, surrounding structure localization, and trajectory application [130]. Blending AR with virtual reality (VR) in the context of mixed reality (MR) offers additional benefits for glioma surgery. While AR can enhance stereoscopy, VR can simulate visuotactile perception during brain manipulation through tissue consistency feedback [131]. The application of advanced methods is growing, with documented examples of spatial drift compensation for markerless spatial registration [132] or MR projection mapping (MRPM) [133]. Figure 5 summarizes some of the most important applications of AI pre- and intraoperatively.

### 4.2. Radiotherapy Planning Optimization

Adjuvant radiotherapy is commonly employed following surgical intervention for gliomas [134], and AI can improve dose calculations and adaptive planning based on patient-specific data. CNN models can predict 3D-dose distribution in intensity-modulated radiation therapy (IMRT) [135] and generate automatic irradiation plans (e.g., deepMTP approach) comparable to kilovoltage CT (kVCT)-based clinical treatment ones [136] to improve tumor targeting while sparing surrounding healthy tissue. Hybrid U-Net models with attention mechanisms outperform conventional modes in both accuracy and speed regarding treatment dose prediction, with the authors recommending further modifications for optimization [137].

Nevertheless, considerable advancements are still required to effectively integrate AI into radiotherapy dose prediction, as significant challenges remain. A key area of focus should be tumor imaging segmentation, as accurately delineating the primary compartments of the lesions can lead to more tailored and effective radiation planning [138]. The maximum potential may lie in CNN tools [139] that can achieve accuracy comparable to experienced physicians [140] through gross tumor volume (GTV) and clinical target volume (CTV) delineation [141]. Although significant steps in dose planning have yet to be made, AI models have been more extensively studied for local relapse prediction post-radiotherapy, something that will be discussed below.

### 4.3. Chemotherapy and Targeted Therapy

Following the strides of AI in surgical and radiotherapy planning, research has introduced great applications in chemotherapeutic response prediction and regimen formulation. Patients with residual gliomas might particularly benefit from novel technologies, according to results on chemotherapy response early prediction using multiparametric MRI-based radiomic models [142]. Differentiating between actual progression and pseudoprogression is one of the hottest topics of temozolomide response prediction and hitherto accomplished from DWI and DSC [143,144], especially when fused with ML [145,146]. A classic tool for treatment response radiological assessment in high-grade gliomas is the Response Assessment in Neuro-Oncology (RANO) criteria [147], whose requirements can be automatically satisfied by algorithms that account for FLAIR hyperintensity and contrast-enhancing tumor volume [148].

Treatment response prediction is a steppingstone for making personalized treatment concrete; AI-powered algorithms can be implemented not just in clinical trials [16,149], but also for personalized chemotherapy regimen personalization in clinical settings. These tools can process vast amounts of patient-specific genetic, molecular, and clinical data (e.g., MGMT methylation status) to suggest schemes that optimally suit each tumor’s individual characteristics, even in limited resource settings [107]. Of course, ML-supported treatment plan tailoring nowadays goes hand in hand with both multi-omic approaches (genomic, transcriptomic, proteomic data) and adverse reaction/toxicity prediction (potential side effect forecast) [150]. Such predictions are increasingly based on subtle inter-cell differences and groups such as, e.g., macrophages concurrently expressing CD163 and N-formyl peptide receptor 3 (FPR3), with prognostic models—in this case the so-called CD163+FPR3+ macrophage-related risk score—thereby developed [151]. These developments lay the foundation for more accurate and precise prediction of targeted therapy response in gliomas.

### 4.4. Emerging Approaches in Immunotherapy

Immunotherapy in gliomas aims to enhance antitumor immune responses through strategies such as immune checkpoint inhibitors (ICIs), chimeric antigen receptor (CAR) T-cell therapy, dendritic cell vaccines, and oncolytic viruses, albeit clinical efficacy remains rather limited due to the immunosuppressive tumor microenvironment [152]. Its characterization, joint with immune profile analysis in cohorts with patient-specific and variable outcomes, has already led to the development of ML models that can predict glioblastoma progression status post-ICI treatment with an accuracy greater than 80% [153]. Nowadays, similar models have even become publicly available for other malignancies: patients can openly access online tools such as the LORIS (Logistic Regression-based Immunotherapy-Response Score) [154] to independently compute their individual ICI response probability across a range of solid tumors [155].

More and more approaches for immunological characterization and response prediction are emerging as neurobiological research delves further into details at a cellular/molecular level. Recent focus was directed towards the role of glycolysis and its association with the tumor microenvironment (TME), as a Chinese team developed a so-called glycolysis-related gene signature (GRS)-TME classifier and identified that GRS^low^/TME^high^ subgroup tumors exhibit a higher response rate to immunotherapy [156]. ML-driven classification of gliomas from large databases into distinct immune subtypes (e.g., IM1-IM4; IMA-IMD) additionally segregates those in richer natural killer (NK) and CD8+ T-cell settings, amplified leucocyte activation and cytotoxicity, etc., to identify patients with favourable genetic mutation profiles and subsequently longer overall survival following immunotherapy [157]. Meticulous fragmentation of available histomolecular classifications is the key to better tailor therapeutic strategies and improve survival, considering that response prediction is in lockstep with survival (prognostic) assessment.

## 5. Prognostic Assessment Using AI

### 5.1. Predicting Patient Survival and Recurrence Risks

Traditional prognostic models such as the Kaplan–Meier estimator and Cox Proportional Hazards (CPH) regression have provided valuable survival estimates but remain inherently limited in their ability to account for non-linear interactions and high-dimensional data complexity in gliomas [158]. AI-driven prognostic models have significantly enhanced survival prediction by integrating multimodal datasets, including imaging-derived radiomics, clinical variables, and molecular biomarkers, with ML and DL algorithms offering a robust alternative to identify nuanced patterns that may not be discernible through conventional statistical approaches [159].

As inferred previously, radiomics-based models enable the extraction of high-throughput quantitative imaging features from MRI, CT, and PET scans to delineate characteristics known to correlate with survival outcomes (such as tumor heterogeneity, texture, and vascularity) with accuracies of up to 98% [160]. The integration of radiomic signatures with clinical variables such as age, the Karnofsky Performance Status (KPS), inflammatory biomarkers, and extent of resection has resulted in improved survival stratification [161,162]. Furthermore, the already-discussed applications of AI in genomics have facilitated the identification of survival-related molecular markers, inducing—but not limited to—IDH mutation, CDKN2A alteration, and MGMT promoter methylation status [163,164], as CNNs trained on histopathological slides have demonstrated their capacity to predict GBM prognosis with high accuracy (over 85%) [165]. Multi-omics AI-powered models incorporating transcriptomic characteristics provide valuable insights [166], although the combination of imaging and non-imaging data has been observed to produce the best results [167].

As ongoing predictive models continue to markedly improve the estimation of the overall survival (OS) and progression-free survival (PFS) in glioma patients, ML algorithms such as SVMs, random survival forests (RSFs) and artificial neural networks (ANNs) continue to demonstrate substantial efficacy in stratifying patients based on recurrence risks and survival probabilities [168]. RSFs handling censored survival data and integrating radiomic, histopathological, and molecular markers are instrumental in capturing complex interrelations among multiple prognostic variables to estimate PFS and outperform traditional regression models in survival stratification and OS prediction, with concordance indexes over 70% [164]. DL architectures including long short-term memory (LSTM) networks have been similarly utilized to model temporal patterns in longitudinal imaging and clinical datasets to enable dynamic predictions and recurrence risk assessments [169].

### 5.2. Monitoring Disease Progression

Another critical application of AI in glioma prognostication involves distinguishing true tumor progression from pseudoprogression and radiation necrosis [170]. Conventional radiological evaluation often struggles to differentiate treatment-induced changes from actual tumor recurrence [171], necessitating AI-based classifier incorporation. Hybrid models integrating multiparametric MRI data with molecular predictors have demonstrated superior accuracy in this context with overall concordance rates as high as 70% and receiver operating characteristic (ROC) analysis accuracies superior to 75% [172], allowing for timely therapeutic adjustments. Furthermore, AI-powered real-time monitoring systems have been developed to dynamically track tumor evolution, thereby enabling clinicians to refine treatment strategies based on personalized risk assessments [16].

Compared to conventional survival estimation methodologies, these prognostic models exhibit superior predictive performance due to their ability to process vast, high-dimensional datasets [173]. Approaches such as CPH regression rely on predefined assumptions about variable interactions and may fail to capture complex survival determinants [174], whereas AI models—and particularly those leveraging DL—excel in feature extraction and predictive analytics [175]; their prediction frameworks integrate imaging, clinical, and molecular data to achieve concordance indices over 80%, significantly outperforming conventional models typically ranging between 65% and 70% [176]. Notably, explainable AI (XAI) techniques such as SHapley Additive exPlanations (SHAP) and Gradient-weighted Class Activation Mapping (Grad-CAM) have been increasingly employed to enhance the interpretability of AI-based prognostic models, addressing the “black-box” nature of DL approaches and fostering clinical acceptance [177], a characteristic that will be discussed in more detail below.

One of the most interesting studies employing multi-parametric MRI voxel-based radiomic features to predict GBM local recurrence regions was published in 2023 in *Cancers* by a Noruego-Hispanic team [178]. The authors utilized four ML-based classifiers to perform voxel-wise extraction of radiomic features from MRI scans, focusing on parameters such as intensity, texture, and shape. Their predictive model demonstrated a mean AUC of 81% with an accuracy of 84% in the external testing cohort in forecasting recurrence regions by associating certain imaging characteristics in the peritumoral zone with a tendency for local recurrence. What discriminates this study from its predecessors is the fact that it is not simply one of the few to narrow its sample down to patients whose contrast-enhancing tumor resection was complete, but the first to validate its findings using external, multi-institutional data and rely both on post- and pre-operative MRI. Discriminating edema from tumor infiltration is crucial to properly adjust patient-specific resection limits and radiotherapy targets, as the peritumoral area is known to host infiltration regions culpable of future local recurrence [179]. Considering how such patterns can often follow discrepancies imperceptible to the human eye and the commonly unpremeditated resection of healthy perilesional tissue intraoperatively [180], one should recognize the importance of focusing on post-operative imaging in such studies, whose methods can be adopted universally and irrespective of acquisition protocols.

### 5.3. Longitudinal Assessment and Follow-Up

The gravity of developing new AI-based protocols—and refining existing ones—is tied to the demand of identifying those patients with true tumor progression; this is especially relevant in cases of “silent” relapse, when the absence of overt symptomatology underscores the importance of follow-up imaging, a process that can—and should—be further enhanced through AI integration. This is necessitated as pseudoprogression is sometimes diagnosed retrospectively—and sometimes conclusively only through invasive tissue sampling—due to the lack of specific pathognomonic MRI features that distinguish it from factual recurrence [181]. Recent large systematic reviews have indeed highlighted the lack of sufficient reliance on conventional MRI techniques in differentiating pseudo-from true progression and accentuated the ability of radiomics to improve pseudoprogression diagnosis accuracy when combined with advanced MRI techniques such as MRS and MR perfusion [182].

Follow-up protocols should not be uniform but tailored based on patient-specific data. Individuals at high risk of local failure (LF) can benefit from stereotactic radiation therapy (SRT) dose escalation, wider clinical target volume (CTV) margins, systemic BBB-penetrative therapy, and stricter follow-up imaging for earlier detection of potential LF [183]. Although studies recognising radiomics as an effective predictor of LF in SRT patients had long been monocentric and lacked external validation [184,185,186], a 2024 paper using data from over 350 patients from the AURORA (A Multicenter Analysis of Stereotactic Radiotherapy to the Resection Cavity of BMs) multicenter study concluded that a combination of clinical and radiomics features is a predictor of LF freedom compared to the sole former, supporting that patients at high risk for LF may benefit from more frequent follow-up imaging and amplified therapy [187].

## 6. Challenges and Limitations of AI in Glioma Management

### 6.1. Data Quality, Availability, and Standardization

Although AI embedment in glioma management is already underway, significant challenges persist. Data heterogeneity is one of the primary issues, as the datasets used for such studies are usually drawn from various sources that include multiple institutions, imaging techniques, and patient demographics. As this diversity can result in incomplete, inconsistent, or even contradictory data when left unresolved, the development of accurate AI models is complicated and perplexing. This data variability also hamstrings reliable generalization across diverse populations and restricts the clinical applicability of both current and novel models.

Another issue arises in acquiring large and annotated datasets that include clinical, imaging, and histological data, as the available datasets are often short of the scale and/or depth required for robust algorithm training. Furthermore, annotated data are labor-intensive with extortionate production costs, and the scarcity of high-quality datasets hinders the development of reliable prediction and recommendation models. The need for standardized protocols warranting uniform data collection procedures, consistent diagnostic criteria, and cohesive data formatting is therefore pressing, while collaborations and data sharing within ethical and legal frameworks across institutions are a prerequisite to build comprehensive datasets that can effectively train existing and upcoming AI models on accurately predicting glioma outcomes. One should also keep in mind that a great proportion of data regarding histological classification and grading of gliomas stems from eras when the molecular profiling of these tumors was not as widespread—or even crucial—, thereby inadvertently contaminating the AI-feeding datasets to a certain extent.

### 6.2. Interpretability and the “Black Box” Nature of AI Models

Despite their radiological imaging analysis potential, DL techniques currently operate partially as complex, opaque systems with an unclear rationale behind their decision-making [188]. Clinicians are still hesitant to fully integrate such systems into their daily practice in high-stakes contexts such as glioma management, due to the lack of complete interpretability and clear understanding of how AI models arrive at conclusions. It is necessary to develop models that do not merely predict outcomes efficiently but also explain the factors contributing to their predictions in detail.

Efforts to improve AI-driven prediction transparency and explainability are luckily—and despite the challenges—already underway. XAI techniques constitute a promising direction and offer interpretable insights into their decision-making process, explaining how they identify key features in radiological scans; this can not only solidify trust in such systems, but also allow the integration of their recommendations into real-life clinical decision-making. Of course, achieving true interpretability in DL models remains challenging due to their ‘black-box’ nature and the involvement of innumerable parameters that complicate the pinpoint of specific prediction-driving features.

### 6.3. Ethical and Legal Implications of AI Integration into Clinical Practice

Challenges revolving around the integration of AI into glioma management extend to its ethical implications, with concerns raised particularly around data privacy [189]. Breaches of privacy or misuse of the large amount of sensitive patient information AI systems rely on (i.e., medical history, genetic profiles, diagnostic results) can compromise patient confidentiality, especially considering how difficult it is to ensure that all data derived from multiple sources remain secure across different platforms and institutions. Ethical concerns also extend to the potential for prediction-affecting bias; AI models might generate inaccurate and/or skewed results that disproportionally represent specific patient groups if certain populations are over- or underrepresented in their training datasets, necessitating strong governance frameworks that not only guarantee individual consent, but maintain transparency in data usage while upholding strict security standards at the same time.

Legal concerns mainly revolve around accountability and regulation, as it is unclear who is responsible when AI systems make incorrect decisions during diagnostic and/or treatment recommendations and result in patient harm [23]. Although the question of legal liability for such errors in complex and varies by jurisdiction, it remains a pressing issue considering the need to responsibly employ AI in clinical settings. Many AI systems in medical practice are inadequately regulated for the time being and lack universal frameworks for safety and effectiveness evaluation. Regulatory bodies ought therefore to establish clear guidelines for AI system approval and monitoring in healthcare to protect patient rights and foster trust in AI applications in glioma management.

## 7. Future Directions and Innovations in AI in Neuro-Oncology

AI has made significant advancements in neuro-oncology, although there are still areas of potential growth. One major improvement would be the incorporation of larger and more diverse datasets to enhance model generalizability and robustness and ensure more accurate predictions (e.g., MGMT methylation and IDH mutations) across different clinical settings [53,190]. Perhaps a key change to greatly improve AI model precision—especially regarding disease progression, overall survival and treatment response—would be the integration of as much multimodal imaging data as possible (i.e., MRI, PET, and radiomics features from the same patient), instead of basing algorithms on unimodal scans [191,192]. Minimizing the reliance on time-consuming and complex training phases through the combination of DL with traditional ML techniques would certainly avail the development process for novel models [193,194], however the expansion of their clinical validation through longitudinal studies might help the most in ensuring that they can be seamlessly integrated into routine diagnostic workflows [148,195].

The future of AI-assisted glioma management becomes more promising as more methods of diagnosis and treatment are explored. Last year, for example, spontaneous Raman spectroscopy was used in the context of a multi-institutional (USA, Finland, Romania, Spain) study to develop classifiers for tumor/non-tumor, IDH1wild-type/IDH1mutated, and methylation subtypes [196]. SVM and Random Forest methods identified key Raman frequencies, which were validated through stimulated Raman spectroscopy and mass spectrometry of glioma cell lines, fabricating the APOLLO (rAman-based PathOLogy of maLignantgliOma) platform. This computational workflow could distinguish tumor tissue from non-tumor, identify Raman peaks related to DNA and proteins more intense in tumors, differentiate between IDH1-mutated and IDH1-wild-type gliomas, and identify specific methylation subtypes, notably distinguishing cytosine-phosphate-guanine island methylator phenotype(G-CIMP)-high from G-CIMP-low within IDH1-mutated tumors.

Advanced AI-driven treatment options will hopefully continue to gain ground in the landscape of gliomas as well; similarly in 2024, a study reported a new ML-based method to identify cell fate determinants (CFD) via GBM cell reprogramming into induced antigen-presenting cells (iDC-APCs) with functions similar to natural dendritic cells (DCs), capable of priming CD8+ T-cells and enhancing antitumor immunity [197]. The approach significantly improved survival in immunocompetent murine models when combined with programmed cell death protein 1 (PD1) decoy immunotherapy and a DC-based GBM vaccine by promoting tumor-specific CD8+ cytotoxic T-lymphocyte (CTL)-mediated killing of GBM cells.

Lastly, Generative AI can play a pivotal role in glioma management by expanding training data diversity and enabling hypothesis-driven experimentation [198]. Diffusion-model–based generative data augmentation can synthesize realistic, diverse MRI phenotypes (e.g., paired T1-weighted and FLAIR images) that help balance underrepresented molecular subgroups (such as IDH-mutant and MGMT methylated tumors) and improve cross-center generalizability. Recent work demonstrates that a generative augmentation approach achieved superior IDH-status classification (internal AUC up to 0.938 and external AUC around 0.83) compared with neuroradiologists, with generated images attaining realism indistinguishable from real scans, particularly for larger or more enhancing lesions [199]. These findings, highlighted in reviews of generative AI for glioma diagnosis, underscore the potential to augment datasets beyond the limits of single-center cohorts and to reduce domain shifts across institutions. Beyond imaging, generative AI could synthesize plausible molecular and radiomic data to fuel novel hypothesis generation and patient-specific treatment simulations, enabling in silico exploration of therapy sequences, dose regimens, and anticipated radiographic trajectories before prospective testing [198,199]. To capitalize on these opportunities, future work should integrate multimodal imaging with molecular data, emphasize rigorous prospective validation, address biases and explainability, and develop clinically translatable pipelines for personalized glioma care.

## 8. Conclusions

AI has enabled the automation of complex tasks and provided potential solutions to long-standing problems in the diagnosis and treatment of gliomas through the application of ML and DL techniques, improving patient outcomes and clinical decision-making. Notwithstanding these developments, significant obstacles to its incorporation into routine practice—such as interpretability, data standardization, and ethical concerns about algorithmic bias and patient privacy—persist. The development of generalizable AI models should therefore become the top priority of future research. This can be achieved by combining larger and more varied datasets, as well as multimodal imaging and molecular data, and by validating predictive frameworks through extensive, prospective, and ideally randomized studies. AI has the potential to revolutionize glioma management and promote a more individualized, effective, and data-driven approach to neuro-oncological care if the present limitations are addressed and the current methodologies are improved.

## Data Availability

No new data were created or analyzed in this study. Data sharing is not applicable to this article.

## Abbreviations

The following abbreviations are used in this manuscript:

^18^F-DOPA	3,4-dihydroxy-6-[^18^F]fluoro-L-phenylalanine
ADC	Apparent diffusion coefficient
AGI	Artificial general intelligence
AI	Artificial intelligence
ANN	Artificial neural network
APOLLO	Raman-based pathology of malignant glioma
AR	Augmented reality
ATRX	Alpha-thalassemia/mental retardation X-linked
AUC	Area under curve
AURORA	A multicenter analysis of stereotactic radiotherapy to the resection cavity of brain metastases
BBB	Blood-brain barrier
BCRP	Breast cancer resistance protein
BraTumIA	Brain tumor image analysis
CAR	Chimeric antigen receptor
CBF	Cerebral blood flow
CDKN2A/B	Cyclin-dependent kinase inhibitor 2A/B
CET	Contrast-enhancing tumor
CFD	Cell fate determinant
Cho	Choline
CIC	Capicua transcriptional repressor
CMRO_2_	Cerebral metabolic rate of oxygen
CNN	Convolutional neural network
CNS	Central nervous system
CPH	Cox proportional hazards
Cr	Creatine
CT	Computed tomography
CTL	Cytotoxic T-lymphocyte
CTV	Clinical target volume
DL	Deep learning
DSC	Dice similarity coefficient
DSC	Dynamic susceptibility contrast
DTI	Diffusion-tensor imaging
DWI	Diffusion-weighted imaging
EGFR	Epidermal growth factor receptor
FDG	^18^F-fluorodeoxyglucose
FLAIR	Fluid-attenuated inversion recovery
FPR3	N-formyl peptide receptor 3
GBM	Glioblastoma multiforme
G-CIMP	Cytosine-phosphate-guanine island methylator phenotype
GPT-3	Generative pre-trained transformer 3
Grad-CAM	Gradient-weighted class activation mapping
GRS	Glycolysis-related gene signature
GTV	Gross tumor volume
HDFT-F	High-definition fiber tractography with sodium fluorescein
HSI	Hyperspectral imaging
HUD	Head-up display
IBM	International Business Machines Corporation
ICI	Immune checkpoint inhibitor
iDC-APC	Induced antigen-presenting cells
IDH1	Isocitrate dehydrogenase 1
IL-6	Interleukin 6
IM	Immune subtype
IMRT	Intensity-modulated radiation therapy
KPS	Karnofsky performance status
kVCT	Kilovoltage computed tomography
Lac	Lactate
LF	Local failure
LORIS	Logistic regression-based immunotherapy-response score
LSTM	Long short-term memory
mAR	Mobile augmented reality
MGMT	O[6]-methylguanine-DNA methyltransferase
MIL	Multi-instance learning
mitoPO_2_	Tissue oxygen saturation
ML	Machine learning
MR	Mixed reality
MRI	Magnetic resonance imaging
MRPM	Magnetic resonance projection mapping
MRS	Magnetic resonance spectroscopy
NAA	N-acetylaspartate
NET2	Non-enhancing T2 hyperintense
NK	Natural killer
NLP	Natural language processing
NLU	Natural language understanding
OEF	Oxygen extraction fraction
OS	Overall survival
PCNSL	Primary central nervous system lymphoma
PD1	Programmed cell death protein 1
PET	Positron emission tomography
PFS	Progression-free survival
P-gp	P-glycoprotein
RANO	Response assessment in neuro-oncology
rCBV	Relative cerebral blood volume
ROC	Receiver operating characteristic
ROI	Region of interest
RSF	Random survival forest
RS-fMRI	Resting-state functional magnetic resonance imaging
SHAP	Shapley additive explanations
SINA	Sina intraoperative neurosurgical assist
SRMA	Systematic review and meta-analysis
SRT	Stereotactic radiation therapy
SVM	Support vector machine
T1GD	T1-weighted gadolinium-enhanced
TCGA	The Cancer Genome Atlas
TCIA	The Cancer Imaging Archive
TERT	Telomerase reverse transcriptase
TERTp	Telomerase reverse transcriptase gene promoter
TME	Tumor microenvironment
TMZ	Temozolomide
TP53	Tumor protein 53
TRIPOD	Transparent reporting of a multivariable prediction model for individual prognosis or diagnosis
TVN	Trans-visible navigator
ViT	Vision transformer
VNIR	Visible and near-infrared
VR	Virtual reality
WHO	World Health Organization
WSI	Whole-slide image
WSL	Weakly supervised learning
XAI	Explainable artificial intelligence
